# Advancements and Challenges in Personalized Therapy for *BRAF*-Mutant Melanoma: A Comprehensive Review

**DOI:** 10.3390/jcm13185409

**Published:** 2024-09-12

**Authors:** Abdulaziz Shebrain, Omer A. Idris, Ali Jawad, Tiantian Zhang, Yan Xing

**Affiliations:** 1 Department of Biological Sciences, Western Michigan University, Kalamazoo, MI 49008, USA; ss.abdulaziz1@gmail.com (A.S.); almaamori94@gmail.com (A.J.); 2 Malate Institute for Medical Research, Malate Inc., P.O. Box 23, Grandville, MI 49468, USA; 3 Toni Stephenson Lymphoma Center, Department of Hematology and Hematopoietic Stem Cell Transplantation, Beckman Research Institute, City of Hope, Duarte, CA 91010, USA; tiantian.zhang@ucf.edu; 4 Department of Medical Oncology and Therapeutics Research, City of Hope, Duarte, CA 91010, USA

**Keywords:** immunotherapy, melanoma, *BRAF* mutation, precision medicine, tumor microenvironment, neoplasm heterogeneity, chimeric antigen receptor T-cell therapy, neoantigens, tumor-infiltrating lymphocytes, lifileucel, biomarker discovery

## Abstract

Over the past several decades, advancements in the treatment of *BRAF*-mutant melanoma have led to the development of *BRAF* inhibitors, *BRAF*/MEK inhibitor combinations, anti-PD-1 therapy, and anti-CTLA4 therapy. Although these therapies have shown substantial efficacy in clinical trials, their sustained effectiveness is often challenged by the tumor microenvironment, which is a highly heterogeneous and complex milieu of immunosuppressive cells that affect tumor progression. The era of personalized medicine holds substantial promise for the tailoring of treatments to individual genetic profiles. However, tumor heterogeneity and immune evasion mechanisms contribute to the resistance to immunotherapy. Despite these challenges, tumor-infiltrating lymphocyte (TIL) therapy, as exemplified by lifileucel, has demonstrated notable efficacy against *BRAF V600*-mutant melanoma. Additionally, early response biomarkers, such as COX-2 and MMP2, along with FDG-PET imaging, offer the potential to improve personalized immunotherapy by predicting patient responses and determining the optimal treatment duration. Future efforts should focus on reducing the T-cell harvesting periods and costs associated with TIL therapy to enhance efficiency and accessibility.

## 1. Introduction

Melanoma is the uncontrolled proliferation of epidermal melanocytes and is the most severe form of skin cancer. Throughout melanoma progression, various molecular changes, such as overactivation of the mitogen-activated protein kinase (MAPK) growth regulatory pathway, can emerge and serve as therapeutic targets [1]. *BRAF* and *NRAS* are two of the most common oncogenes involved in melanoma [2]. The *BRAF* protein, encoded by the *BRAF* gene, is involved in the MAPK pathway, which comprises a chain of intracellular proteins that regulate cell growth, apoptosis, and differentiation [3].

*BRAF* mutations are present in approximately 50% of the patients with metastatic melanoma. The two most common forms of *BRAF* mutations among patients with *BRAF*-mutant melanoma are the *V600E* and *V600K* mutations, with the former found in 70–90% of these patients. Most *BRAF* mutations affect the kinase domain of the *BRAF* protein, resulting in constitutive activity and elevated MAPK signaling, which collectively promote malignant development in vitro [4].

Multiple therapies have been developed to treat *BRAF*-mutant melanomas through the characterization and identification of numerous *BRAF* mutations. According to the guidelines of the European Society for Medical Oncology (ESMO), thorough molecular testing is required for patients with melanoma, and mutation testing for actionable mutations is necessary for patients with resectable or unresectable stage III or IV melanoma. Moreover, the ESMO guidelines require *BRAF* testing in patients with melanoma, which can lead to further genetic sequencing [5]. The results of such analyses can facilitate the development of targeted treatments for *BRAF*-mutant melanomas. Before the approval of the first *BRAF* inhibitor, chemotherapy was the standard of care for patients with advanced or metastatic melanoma. However, with the implementation of *BRAF* inhibition, the median survival of treated patients increased from six months with chemotherapy to 25.9–33.6 months. In the adjuvant setting, targeted therapies for melanoma substantially reduced the risk of relapse by 53% compared to placebo, leading to the approval of *BRAF* plus MEK inhibitors as a treatment option for stage III melanoma [6]. Various combinations of *BRAF*/MEK inhibitors have been approved for patients with *BRAF V600*-mutant melanoma [7]. For instance, a combination of the *BRAF* inhibitor da*BRAF*enib and the MEK inhibitor trametinib has been used to treat solid metastatic tumors harboring *BRAF V600E* mutations [8]. Immunotherapy is also a treatment option for *BRAF*-mutant melanoma that includes treatments involving interleukin 2, interferons, and programmed cell death protein 1 (PD-1) [8]. Anti-programmed death 1 (PD-1) antibodies, either alone or in combination with an anti-cytotoxic T lymphocyte-associated antigen-4 (CTLA-4) antibody, have been used in the treatment of *BRAF*-mutant melanoma. Targeted therapy for *BRAF*-mutant melanoma generally achieves excellent tolerance with low toxicity; however, resistance to therapy typically emerges after 12–18 months of treatment. Additionally, although immunotherapy has led to durable responses in these patients, the combination of the anti-PD-1 antibody nivolumab and the anti-CTLA-4 antibody ipilimumab has produced high-grade toxicities in over 50% of the patients [9].

Given the emergence of adverse events associated with immunotherapy, precision medicine represents a promising sphere of immunotherapy for personalized treatment of *BRAF*-mutant melanoma. Routine clinical tests tailored for patients can involve genomic and molecular profiling using next-generation sequencing technologies. Cancer immunotherapy represents a potential approach for maximizing the utility of genomic sequencing in cancer patients using neoantigens, which are cancer-specific antigens derived from somatic mutations. Through successful neoantigen targeting, personalized immunotherapies such as cancer vaccines and T-cell receptor-engineered T-cell therapy can be developed for larger groups of patients with cancer [10]. This review explores the advancements and difficulties in personalized immunotherapy for *BRAF*-mutant melanoma. Personalized therapy for *BRAF*-mutant melanoma represents a significant advancement over traditional treatment approaches by tailoring interventions based on individual genetic profiles. Personalized immunotherapy specifically aims to target unique molecular and genetic features of the tumor, providing a more precise and effective treatment compared to standard, one-size-fits-all approaches.

Standard treatments typically include broad-spectrum therapies that are not tailored to the individual’s specific tumor characteristics. In contrast, personalized immunotherapy leverages detailed genetic and molecular analyses to select treatments that are expected to be most effective based on the patient’s unique tumor profile. This approach not only aims to enhance therapeutic efficacy but also to minimize unnecessary side effects associated with less targeted therapies. The distinction between personalized immunotherapy and standard treatments is critical in understanding how personalized strategies offer improvements over conventional methods and address the specific challenges posed by the tumor microenvironment (TME) [11,12,13].

## 2. Tumor Heterogeneity and Its Implications for *BRAF*-Mutant Melanoma

### 2.1. Introduction to Tumor Heterogeneity and the Tumor Microenvironment

Tumors can form from multiple genetically unique cell populations, resulting in tumor heterogeneity, which defines the cellular diversity of the tumor microenvironment (TME). The TME harbors an assemblage of immune cells, including cancer-associated fibroblasts and endothelial cells, that affect tumor progression and response to therapy [14]. The adaptation of the TME to therapy promotes therapy-resistant residual disease cells [15]. In approximately 4–25% of patients, melanoma has been correlated with the inter-tumor heterogeneity of *BRAF* [16]. Genomic instability is a critical contributor to intratumoral heterogeneity. According to the clonal evolution/selection hypothesis, an induced genetic alteration in a previously non-malignant cell gives rise to neoplastic proliferation, driven by the selective growth advantage of the genetic change. The resulting genomic instability of the growing tumor population generates additional diversity, which can be influenced by evolutionary selection pressures that yield increasingly heterogeneous subpopulations. Within the framework of this hypothesis, two major evolutionary patterns exist: linear and branched. Linear evolution is characterized by successive mutations that are advantageous for growth and survival. In this model, sequential clones with advantageous genetic changes outcompete the ancestral clones. Through branched evolution, several subclonal tumor cell populations arise from a common ancestor [17]. In a previous meta-analysis, a clinically significant discrepancy in *BRAF* status was observed between metastatic and primary metastatic melanoma lesions. This discrepancy is most likely explained by the true intratumoral heterogeneity, a mechanism supported by the polyclonality model. The polyclonality framework suggests that primary and metastatic melanoma lesions can harbor *BRAF*-wild-type and *BRAF*-mutated subclone populations capable of metastasis [18]. In a case report of a 49-year-old Japanese woman diagnosed with metastatic *BRAF*-mutant melanoma, Sanger sequencing revealed *BRAF* heterogeneity, in which the status of the primary tumor and skin metastatic lesions were *BRAF V600E* and *BRAF* wild, respectively, suggesting that melanoma of the *BRAF* genotype is occasionally heterogeneous [19]. In a large cohort study, tumor samples from 60 patients with melanoma treated with immune checkpoint blockade or targeted therapy were analyzed. Among the patient samples, 36% had detectable mutations in *BRAF*, and mutations in NRAS and *BRAF* were maintained across synchronous metastases. Based on the radiological assessment, heterogeneity was observed in the responses of most patients to treatment. Through genomic and immune profiling, synchronous melanoma metastases were found to have significant genomic and immune heterogeneity in all patients, particularly in their T-cell repertoires. Moreover, divergent immune profiles within the patient cohort correlated with variability in therapy responses. However, immune and genomic factors were found to be closely related based on the positive correlation between mutational burden and CD8+ T-cell infiltration and the greater response of clonal T cells [20].

Tumor heterogeneity is one of the main barriers to the development of effective personalized medicine for cancer treatment. Sufficient characterization of tumor cell populations is necessary for effective cancer therapy. Intratumoral heterogeneity complicates this approach by rendering singular biopsies insufficient for the complete and highly accurate evaluation of the tumor landscape [21]. Over the course of anti-tumor treatment, cells and all immune components of the TME respond to stressful and sustained anti-tumor agents by implementing an adaptive mechanism that creates a novel homeostasis for the tumor and its immune compartment. Because of their intrinsic heterogeneity, cells in the TME exhibit differential responses to therapeutic treatments [22]. Despite being initially effective, therapies inhibiting *BRAF V600E* are often limited by the development of drug-resistant tumor subpopulations in the TME. An empirically supported mechanism of this resistance involves the constitutive production of fibroblast growth factor-2 in tumors and the resulting activation of inflammatory factors and cytokine production by B cells. Consequent crosstalk results in melanoma heterogeneity [23].

Multiple studies have assessed how TME enables resistance to immunotherapy in patients with melanoma. In one study, the intrinsic resistance of melanoma cells to immune checkpoint blockade was comprehensively explored by structurally analyzing the treatment-naïve melanoma ecosystem. The TME supports the development of complex melanoma transcriptomes. In addition, melanoma cells in the mesenchymal-like state (MES), which are known to promote resistance to targeted therapy, were present in substantial numbers in immune checkpoint-blocked (ICB) non-responders. Transcription factor 4 (TCF4), a master MES regulator, suppresses antigen and melanocyte presentation transcriptional programs, indicating that TCF4 is a potential genetic or pharmacological target to increase the sensitivity and immunogenicity of MES cells for targeted therapy and immune checkpoint blockade [24]. In another study, changes in the TME that confer resistance to immune checkpoint inhibitors during the treatment of metastatic melanoma were explored. A notable proportion of patients with advanced melanoma fail to respond to or develop cancer after an initial response to various immunotherapies. Numerous potential causes for this resistance have been identified, including changes in interferon signaling, activation of immunosuppressive pathways, and downregulation of immune checkpoint ligands. In one study, mice having *BRAF V600E*-mutant melanoma with or without deletion of phosphatase and tensin homolog (PTEN) and active beta-catenin were involved in molecular profiling. The results of the study indicated that suppression of T-cell gene expression as a method of immune evasion can be impacted by activation of the WNT/beta-catenin pathway [25].

### 2.2. Genetic and Immune Interactions

In approximately 44% of melanomas, *BRAF* mutations are accompanied by PTEN loss, an outcome that promotes high levels of immunosuppressive elements such as myeloid-derived suppressor cells and low levels of natural killer (NK) and cytotoxic T cells. Moreover, melanoma cells with a loss of PTEN inhibit anti-tumor T-cell activity, thereby preventing a response to immunotherapy. PTEN also inhibits immunosuppressive cytokine production by negatively regulating the PI3K and signal transducer and activator of transcription 3 [26]. In a comprehensive study of 124 human melanoma cases that included mutations in *BRAF* and NRAS, whole-genome and targeted sequencing analyses of tumor samples revealed that variable responses to treatment among samples from the same patient may be attributable to differential mutation frequencies and sample-specific genetic modifications [27].

### 2.3. Metastatic Heterogeneity and Immune Evasion in BRAF-Mutant Melanoma

Metastatic melanoma is characterized by significant genomic heterogeneity and immune evasion mechanisms, which complicate effective treatment. Whole-exome sequencing of metastatic melanoma deposits has revealed substantial genomic diversity, suggesting that metastases often arise from various subclonal populations within the primary tumor. This heterogeneity can result from branch mutations induced by ultraviolet radiation or selective gains of mutant *BRAF* alleles during early tumor evolution, contributing to differential metastasis and immune evasion [28,29]. In some cases, metastatic clones diverge significantly from dominant populations within primary tumors, complicating treatment strategies [29]. Tumors with high heterogeneity are associated with reduced immune cell infiltration and impaired immunomodulatory gene expression, which leads to lower anti-tumor immune responses, including declines in CD8+ T cells, M1-like macrophages, and T follicular helper cells, while promoting tumorigenic M2-like macrophages [30].

### 2.4. Mechanisms of Drug Resistance in BRAF V600E-Mutant Melanoma

**Figure 1:**
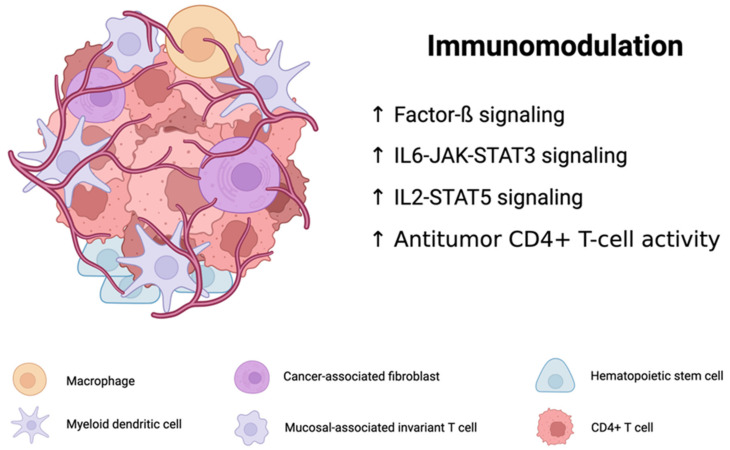
Components and immunomodulatory effects of the *BRAF*-mutant melanoma tumor microenvironment (created with BioRender.com). This figure illustrates the complex interplay of immune cells and signaling pathways within the tumor microenvironment of *BRAF*-mutant melanoma. Key components include CD4+ T cells that enhance anti-tumor activity, macrophages that influence tumor growth and metastasis, myeloid dendritic cells that modulate T-cell responses, and cancer-associated fibroblasts (CAFs) that promote immune evasion. Hematopoietic stem cells and mucosal-associated invariant T cells (MAIT) contribute to shaping the immune landscape, collectively impacting anti-tumor immunity and treatment outcomes.

Mechanisms of resistance in *BRAF*-mutant melanoma are closely linked to tumor heterogeneity and the tumor microenvironment (TME). Resistance to *BRAF V600E* inhibitors frequently arises from various adaptive responses within the TME, including the production of fibroblast growth factor-2, which stimulates the production of inflammatory factors and cytokines by B cells, fostering melanoma heterogeneity [23]. The TME also supports the development of complex melanoma transcriptomes that enable resistance to therapies, such as immune checkpoint blockade, which is further complicated by the intrinsic genetic diversity and adaptive capabilities of melanoma cells [23] (Figure 1).

## 3. Current Approaches to Personalized Therapy for Patients with B-RAF Mutant Melanoma

### 3.1. Tumor-Infiltrating Lymphocyte (TIL) Therapy

TIL therapy has emerged as a personalized immunotherapeutic approach for patients with *BRAF*-mutant melanoma. Adoptive cell therapy using TILs is another therapeutic option for metastatic melanoma. In cancer therapy, a portion of the tumor is isolated by ex vivo expansion, which removes TILs from the TME and prevents intratumoral regulatory T cells from exerting immunosuppressive effects. Rejuvenated TILs can be infused into patients from whom they are obtained [31]. Lifileucel is an unmodified, autologous TIL infusion that has shown effective results in a multicenter, international, phase II multicohort study that included patients with *BRAF V600*-mutant melanoma. Owing to its clinical effectiveness, lifileucel was the first TIL therapy approved for the treatment of advanced melanoma [32]. Lifileucel, an FDA-approved tumor-infiltrating lymphocyte therapy, has demonstrated promising outcomes in treating advanced-stage melanoma [33]. This therapy utilizes autologous tumor-infiltrating lymphocytes (TILs) that are expanded ex vivo and subsequently reintroduced into patients. Lifileucel has demonstrated improved response rates in patients with advanced or unresectable melanoma who have progressed following treatment with immune checkpoint inhibitors and, when applicable, *BRAF*/MEK inhibitors [34]. Studies have indicated that lifileucel can lead to durable responses and potentially clinically meaningful activity in patients with advanced melanoma [35]. In a phase 2 clinical trial, the objective response rate (ORR) for lifileucel was 36.4%, with a median duration of response of 16.8 months. Some patients achieved ongoing responses lasting over two years, demonstrating its potential for long-term disease control in heavily pretreated populations [36,37]. Lifileucel has shown efficacy even in patients who have not responded to multiple lines of prior therapies, including checkpoint inhibitors and *BRAF*/MEK inhibitors [38]. Furthermore, lifileucel has been found to be safe and effective in patients with PD-1 refractory melanoma, as demonstrated in clinical trials [39]. The therapy has also been evaluated in patients who have progressed on immune checkpoint inhibitors and targeted therapies, demonstrating a favorable safety profile and producing durable responses across different patient subgroups, supporting its potential benefit for a broad population of melanoma patients [40]. Lifileucel has demonstrated encouraging efficacy in phase II trials for patients with previously treated metastatic melanoma, as well as recurrent, metastatic, or persistent cervical cancer.

### 3.2. CAR T-Cell Therapy

Chimeric antigen receptor (CAR) T-cell therapy is a cutting-edge immunotherapy approach for treating metastatic melanoma with the aim of harnessing the power of the immune system to target and eliminate cancer cells specifically. CAR T-cell therapy involves genetic modification of patients’ T-lymphocytes to express synthetic receptors known as CARs, which are designed to recognize tumor-specific antigens. Upon recognition of the target antigen, CAR-T cells are activated, leading to the destruction of malignant cells through various effector mechanisms, including cytokine release, cytotoxicity, and the recruitment of other immune cells [41,42] Numerous preclinical studies have elucidated the mechanisms underlying CAR T-cell therapy’s anti-tumor activity, highlighting its potential to induce robust and durable responses in melanoma patients.

In clinical settings, CAR T-cell therapy has shown promise in early-phase clinical trials for metastatic melanoma, with several investigational studies demonstrating encouraging results in terms of safety, efficacy, and feasibility [43,44]. Notably, recent advancements in CAR T-cell engineering, such as the incorporation of novel costimulatory domains and the use of dual-targeting CARs, have further enhanced the therapeutic potential of CAR T-cell therapy for melanoma [45,46]. Despite these advancements, significant challenges persist in the clinical translation of CAR T-cell therapy for treating melanoma. These challenges encompass various aspects, including identification of optimal target antigens, mitigation of off-target effects, optimization of CAR T-cell trafficking and persistence within the tumor microenvironment, and management of immune-related toxicities [47,48]. Moreover, the high cost and logistical complexities associated with CAR T-cell manufacturing and administration pose additional hurdles to their widespread clinical implementation. CAR T-cell therapy holds immense promise as a personalized and targeted treatment approach for metastatic melanoma, and further research efforts are warranted to overcome existing challenges and maximize its therapeutic potential in clinical practice.

### 3.3. Biomarker Detection

Identification of early response biomarkers for advanced melanoma therapies is crucial for assessing the effectiveness of immunotherapy against melanoma. Several biomarkers have been proposed for use in immunotherapy and targeted treatments, with varying degrees of clinical validation. For instance, circulating tumor DNA (ctDNA), particularly *BRAF V600E* ctDNA, has shown promise as a dynamic biomarker for monitoring treatment response, with a decline in its levels often correlating with positive imaging-based response detection in patients undergoing immunotherapy or targeted treatments, making it a clinically validated tool in practice [49]. In contrast, several biomarker candidates are still under investigation. For BRAF-mutant melanoma, lowered levels of extracellular vesicle-melanoma membrane-bound proteins such as LNGFR, MCAM, MCSP, and ERBB3 have been correlated with patient responses to BRAF/MEK inhibitors; however, further validation is required before these can be routinely implemented in clinical practice [50]. Similarly, extracellular vesicle-PD-1 has shown potential as a dynamic biomarker of patient response to melanoma immunotherapy, though it remains in need of further clinical validation to confirm its effectiveness and practicality in routine settings [49]. Matrix metalloproteinase 2 (MMP2) has been identified as a biomarker associated with BRAF mutations and is negatively correlated with survival in patients with BRAF-mutant melanoma, yet its application in clinical practice is limited [51]. Additionally, while genetic ablation of prostaglandin E synthases or cyclooxygenases (COXs) in melanoma cells of *BRAF V600E* mice suggests COX-2 levels in human melanoma could serve as predictive biomarkers for therapies such as checkpoint blockade inhibitors, this finding is primarily based on preclinical data, and its clinical applicability needs further exploration [52]. Metabolic imaging techniques, including deuterium metabolic imaging and 18F-labeled fluorodeoxyglucose–positron emission tomography (FDG-PET), have demonstrated potential in assessing patient response to various therapies, although their role as routine biomarkers is still evolving [53]. Finally, adoptive cell therapy using tumor-infiltrating lymphocytes (TILs) represents a promising personalized immunotherapy strategy, but its broader implementation is constrained by logistical and cost-related factors [31]. While some biomarkers, such as ctDNA, are already clinically validated and used in practice, others remain theoretical or require additional validation before they can be widely adopted in clinical settings.

### 3.4. Targeted Therapy

**Figure 2:**
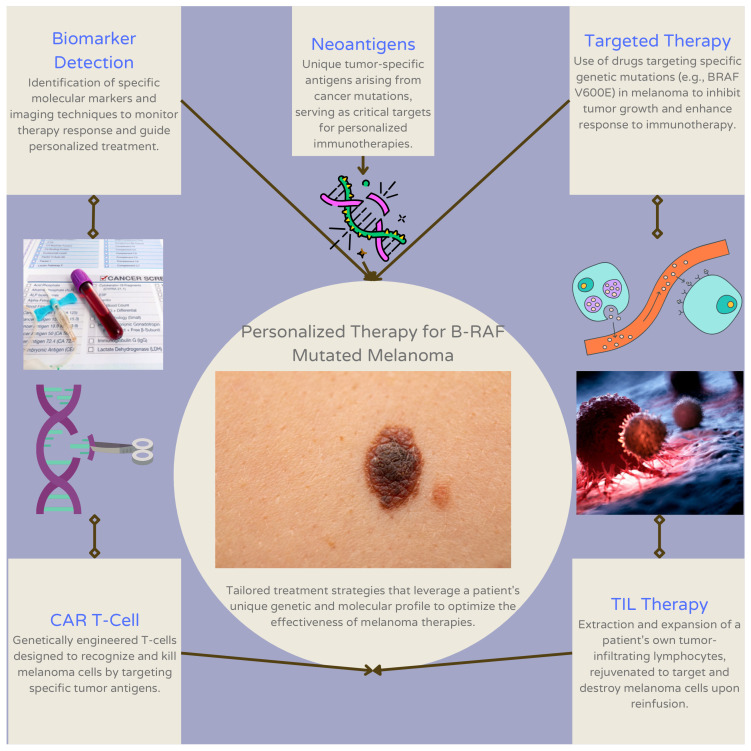
Visual representation of personalized therapy pathways in *BRAF*-mutant melanoma.

Immunotherapy resistance in patients with melanoma demonstrates the need to tailor such therapies to the genomic landscape of individual patients [54]. In a retrospective review of 140 patients diagnosed with advanced melanoma, patients with advanced *BRAF*-mutant melanoma who were treated with first-line immunotherapy showed significantly longer progression-free survival and overall survival (OS) in comparison to patients treated with first-line *BRAF*/MEK inhibitors. However, the latter treatment yielded a higher overall response rate relative to patients treated with first-line immunotherapy [55]. Combining targeted therapy and immunotherapy in patients with *BRAF*-mutant melanoma has yielded limited effectiveness. One phase 3 trial showed a significant improvement in progression-free survival (PFS) through the addition of anti-PD-L1 to *BRAF* and MEK inhibitors (i.e., vemurafenib and cobimetinib, with or without pembrolizumab). However, in a phase 2 trial, combining an anti-PD-1 with targeted therapy did not demonstrate statistically significant improvement in PFS, and the OS rates were similar between the two arms used for administration of the combinatorial treatment (i.e., da*BRAF*enib and trametinib, with or without pembrolizumab) [3]. Responses to personalized immunotherapy can be improved by the supplemental administration of MEK inhibitors, which can promote tumor-associated antigen presentation before or during antigen-specific and immune checkpoint-inhibiting immunotherapies [56]. In a prospective cohort study of 83 patients diagnosed with metastatic melanoma, the levels of the von Willebrand factor antigen underwent differential evolution in patients receiving immunotherapy and served as a prognostic factor for therapy [57]. In a study that reviewed the somatic mutation profiles of 467 melanoma patients, *BRAF* was identified as one of the most mutated genes within the patient cohort. Tumor mutation burden is positively associated with prognosis and immune infiltration, thereby indicating its role in predicting patient response to immunotherapy [58]. The combination of atezolizumab, cobimetinib, and vemurafenib (vem-cobi-atezo) has demonstrated potential therapeutic efficacy in the treatment of various cancers as per a phase III clinical trial [59]. Atezolizumab, a PD-L1-blocking antibody, interferes with the PD-L1 ligand’s binding to its receptors, PD-1 and B7.1 [60]. Cobimetinib, meanwhile, is a MEK inhibitor that targets the MEK pathway in cancer cells [61]. In HIV therapy, cobicistat is used as a pharmacoenhancer to enhance the intestinal absorption of various drugs, including HIV protease inhibitors [62]. Results of this clinical trial showed that the combination of atezolizumab and targeted therapy significantly improved progression-free survival (15.1 months) of cancer patients compared to the control group (10.6 months). Common side effects included elevated blood creatinine phosphokinase levels, diarrhea, rash, and other symptoms. The study concluded that addition of atezolizumab to targeted therapy was safe, well-tolerated, and effective in prolonging progression-free survival in this patient population. Figure 2 visually represents the personalized therapy pathways for *BRAF*-mutant melanoma, while Table 1 provides a summary of these approaches, including tumor-infiltrating lymphocyte (TIL) therapy, T-cell therapy, biomarker detection, targeted therapy, and neoantigen-based strategies. Both illustrate the range of current strategies aimed at optimizing treatment effectiveness by leveraging specific genetic and molecular markers unique to each patient.

This figure outlines personalized therapy pathways in *BRAF*-mutant melanoma. Key components include biomarker detection for identifying specific molecular markers and monitoring therapy response, and neoantigens, which are unique tumor-specific antigens that serve as critical targets for personalized immunotherapies. Targeted therapy focuses on inhibiting tumor growth by targeting specific genetic mutations (e.g., *BRAF V600E*) to enhance immunotherapy response. CAR T-cell therapy involves genetically engineered T-cells designed to recognize and kill melanoma cells, while TIL therapy uses the extraction and expansion of a patient’s own tumor-infiltrating lymphocytes to target and destroy melanoma cells.

### 3.5. Neoantigens

A high burden of tumoral mutation is known to give rise to tumor neoantigens, which enhance the efficacy of cancer immunotherapy via interactions with antigen-specific T-cell receptors. Such recognition induces a particular anti-tumor immune response [3]. Among 27 patients treated with adoptive T-cell therapy (ACT) for stage IV melanoma after failing prior immunotherapies (i.e., anti-CTLA-4 and/or intravenous IL-2), higher predicted neoantigen and mutational loads were associated with clinical benefit. This is a critical finding considering that 50–60% of melanoma patients who have been treated with ACT showed no clinical benefit. Given that melanoma has one of the highest average mutational loads among all tumor types, this finding likely indicates an elevated likelihood of neoantigen production capable of stimulating T-cell reactivity [63]. In a clinical study, personalized neoantigen vaccines were developed for six patients with melanoma. Each vaccine targeted up to 20 predicted, personal tumoral neoantigens. Polyfunctional CD8+ and CD4+ T cells were activated through vaccination and were found to target 15 and 58, respectively, of the 97 specific neoantigens used in the patient cohort. Four of the six patients showed no recurrence 25 months after neoantigen vaccination. The remaining two patients showed recurrent disease that showed complete tumoral regression and neoantigen-specific T-cell expansion after anti-PD-1 therapy [64].

## 4. Discussion

### 4.1. Challenges and Opportunities

#### 4.1.1. Challenges

Personalized immunotherapies for *BRAF*-mutant melanomas present several challenges (Table 2). An in vitro study exploring how combined *BRAF* and MEK inhibitors affect adoptive CAR-T cell therapy, combined with da*BRAF*enib and trametinib, was found to hinder CAR-T cell functionality to a lesser extent than combined vemurafenib and cobimetinib by inhibiting the cytolytic capacity [64]. In addition to CAR-T therapy, TIL therapy presents challenges that include delayed patient intervention because of the time needed to harvest and generate viable T-cell populations, with typical periods ranging from approximately three weeks to three months in duration [32]. Moreover, TIL therapy involves a costly manufacturing process that limits patient access to treatment [65]. T-cell receptor-engineered T-cells designed to target antigens common to melanocytes and melanoma cells can also cause off-tumor and on-target toxicities [66]. Additionally, PD-L1 expression lacks biomarker potential for optimizing treatment selection (i.e., anti-PD-1 therapy, monotherapy, or combination immunotherapy) in patients with melanoma [67].

#### 4.1.2. Opportunities

Although personalized immunotherapies for *BRAF*-mutant melanoma present challenges in terms of efficacy and safety, these treatments offer numerous clinical opportunities. In a phase 1 trial that tested the efficacy of GD2-specific CAR-T cell therapy in 14 patients with *BRAF V600*-mutant metastatic melanoma (five of whom received standard, concurrent da*BRAF*enib, and trametinib), 93% of the prepared CAR-T cell products administered to 12 of the 14 patients were acceptable. CAR-T cell expression varied from 21% to 69%. Within the cohort of 12 enrolled patients, no dose-limiting toxicities were observed, and 83% of the patients experienced at least one treatment-emergent adverse event (AE), none of which exceeded grade 2 severity. These AEs included rashes (50%) and diarrhea (33%) [63]. Additionally, to overcome the extensive temporal length of T-cell harvesting, TIL therapy could potentially be improved by freezing tumor sample TILs or by manufacturing intermediates that are readily available for finalizing TIL preparation for clinical TIL administration. However, the clinical implications of this therapeutic approach remain unclear [32]. Additionally, as a personalized approach to safely discontinue anti-PD-1 monotherapy in patients with melanoma, ^18^fluorodeoxyglucose (^18^FDG)-PET/CT scanning has shown promise, yielding results that warrant validation [68]. The combination of TIL therapy with targeted therapy remains an area for future research because of its potential to induce sustained, polyclonal, and anti-tumor T-cell responses through antigen dispersal [69]. Imaging biomarkers also have notable potential for optimizing the treatment of malignant melanoma. PET/CT with ^18^F-FDG has been effectively used to stage advanced malignant melanomas, and multiple parameters derived from PET scans have prognostic value for patients assigned to receive immunotherapy and targeted therapy [70]. Next-generation sequencing can provide a similar conclusion through the precise characterization of melanoma subtypes and elucidation of the points of immune checkpoint inhibitor failure in patients [71]. Table 2 outlines the challenges and opportunities associated with various approaches to personalized immunotherapy for *BRAF*-mutant melanoma. For CAR-T cell therapy, challenges include treatment-related adverse events and limitations in combination with certain targeted therapies, while opportunities lie in exploring new combination regimens. TIL therapy faces challenges such as lengthy cell harvesting periods, high manufacturing costs, and toxicities, with opportunities in optimizing efficiency through cryopreservation and alternative IL-2 delivery methods. Limited literature exists on artificial intelligence applications in 18FDG-PET/CT scanning, but various PET parameters show prognostic value for metastatic melanoma patients undergoing immunotherapy.

**Table 2:** Challenges and opportunities in potential approaches to personalized immunotherapy for *BRAF*-mutant melanoma.

Treatment Approach	Challenges	Opportunities	References
CAR-T cell therapy	Emergence of treatment-related adverse events, hindrance of CAR-T cell functionality in combination with da*BRAF*enib and trametinib	Exploring patient responses to other concurrent CAR-T cell and combined targeted therapy regimens	[63,64]
TIL therapy	Extensive duration of T-cell harvesting periods, cost of manufacturing, toxicities	Freezing tumor-sample TILs for improved efficiency To mitigate the toxicity associated with IL-2 therapy, alternative strategies have been explored. One such approach involves using mesenchymal stem cells (MSCs) as a vehicle for targeted delivery of IL-2 to reduce systemic toxicity [68] Genetically engineered IL-2 variants have been developed to potentially reduce systemic toxicity while maintaining efficacy in immunotherapy for solid tumors	[31,61,62,72,73,74]
^18^FDG-PET/CT scanning	Relative limitation of literature regarding artificial intelligence-based techniques in ^18^FDG-PET/CT scanning for diagnosing melanoma	Multiple PET parameters hold prognostic value for patients with metastatic melanoma undergoing immunotherapy	[70]

CAR, chimeric antigen receptor; FDG, fluorodeoxyglucose; TIL, tumor-infiltrating lymphocytes.

### 4.2. Dynamic and Adaptations within the Tumor Microenvironment

#### 4.2.1. Impact of Tumor Microenvironment (TME)

The TME contains a highly complex and heterogeneous group of immunosuppressive cell populations such as cancer-associated fibroblasts, regulatory T cells, regulatory B cells, and vascular endothelial cells [75]. The TME also contains multiple T-cell populations that influence tumor formation. Cytotoxic T cells (CD8+) influence tumorigenesis by inducing the destruction of tumor cells and detecting abnormal cancer cell antigens. Moreover, the presence of CD8+ cells in the TME is frequently correlated with favorable patient prognosis [67]. The TME contains an assortment of innate immune cells, primarily macrophages, NK cells, dendritic cells, bone marrow-derived suppressor cells, and neutrophils. These immune cells exhibit anti-tumor or pro-tumor activities through the release of cytokines and chemokines [76]. In addition to immune cells, stromal cells are found in the TME and substantially affect tumor metastasis, development, treatment resistance, and immune evasion. Tumor-associated stromal cells communicate with various components of the TME through paracrine or cell-to-cell interactions involving cytokines and mediators. The effect of stromal cells on tumorigenesis illustrates their importance in improving the efficacy of cancer treatment [77]. Furthermore, within the TME, competition for metabolically necessary components may occur between the tumor and immune cells, consequently inhibiting the function of immune cells in the TME (Figure 1) [78].

#### 4.2.2. Tumor Microenvironment (TME) Adaptation

The TME harbors an assemblage of immune cells, including cancer-associated fibroblasts and endothelial cells, which affect tumor progression and the response to therapy [14]. The adaptation of the TME to therapy promotes therapy-resistant residual disease cells [15]. Over the course of anti-tumor treatment, cells and all immune components of the TME respond to stressful and sustained anti-tumor agents by implementing an adaptive mechanism that creates a novel homeostasis for the tumor and its immune compartment. Because of their intrinsic heterogeneity, cells in the TME exhibit differential responses to therapeutic treatments [22].

Multiple features of the TME in *BRAF*-mutant melanomas have been characterized. In a study of patients with *BRAF*-mutant and *BRAF*-wild-type melanoma, *BRAF*-mutant tumors exhibited a higher degree of differential gene expression in comparison to *BRAF*-wild-type tumors of metastatic samples. Moreover, the *BRAF*-mutant tumors contained higher magnitudes of inflammatory response, as well as factor-ß, IL6-JAK-STAT3, and IL2-STAT5 signaling. Such differences in gene expression and signaling pathways between tumor types were more pronounced in the Cancer Genome Atlas skin cutaneous melanoma (SKCM) cohort when melanoma developed into regional metastasis. The *BRAF*-mutant tumor samples also contained elevated levels of macrophages, mucosal-associated invariant T cells, cancer-associated fibroblasts, hematopoietic stem cells, common myeloid progenitors, myeloid dendritic cells, and CD4+ T cells compared to the *BRAF*-wild-type tumor samples. Additionally, compared with their wild-type counterparts, *BRAF*-mutant tumors in the primary SKCM cohort were found to have reduced levels of neutrophils, NK cells, M2 macrophages, B cells, monocytes, and T cells. Overall, bulk RNA-seq data revealed elevated levels of B cells in samples of metastatic *BRAF*-mutant melanoma and CD8+ T cells in wild-type metastatic samples [79]. In a study on innate immune cell infiltration in 385 primary tumors and 96 pairs of metastases, elevated counts of plasmacytoid dendritic cells were found in *BRAF V600E*-positive primary tumors and metastases relative to *BRAF V600E*-negative tumors [80].

The distinct features of the *BRAF*-mutant TME enable its immunomodulatory effects. Through the elevated levels of CD4+ T cells previously found in *BRAF*-mutant tumor samples, the *BRAF*-mutant TME can promote anti-tumor activity through the direct or indirect targeting of tumor cells by CD4+ T cells, which also supports the cytotoxic effects of CD8 + T cells on tumor cells [62,64]. Given the supportive role of CD8+ T cells in cancer immunotherapy, a decline in CD8+ T-cell signatures, as found in a cohort of patients with cutaneous metastatic melanoma, whose collective incidence of tumoral *BRAF* mutations was 21.3%, could independently act as a barrier to immunotherapy [81,82,83,84,85].

### 4.3. Future Directions

The treatment landscape for *BRAF*-mutant melanoma is rapidly evolving, particularly with the development of next-generation *BRAF* inhibitors aimed at overcoming resistance mechanisms inherent to current therapies. Recent preclinical studies have demonstrated that these novel inhibitors can effectively target both monomeric and dimeric *BRAF* mutants, thereby enhancing the inhibition of the MAPK pathway even in the presence of secondary mutations that confer resistance to existing treatments [86]. This advancement is crucial, as resistance to *BRAF*-targeted therapies remains a significant challenge, necessitating innovative approaches to improve patient outcomes [87]. In addition to targeting *BRAF*, the integration of therapies that address the tumor microenvironment (TME) presents a promising avenue for enhancing treatment efficacy. For instance, therapies aimed at tumor-associated macrophages (TAMs) and cancer-associated fibroblasts (CAFs) could potentially disrupt the immunosuppressive environment that facilitates tumor progression and immune evasion [88]. Such strategies may synergize with existing immunotherapies, including immune checkpoint inhibitors, to improve response rates and overall survival in patients with *BRAF*-mutant melanoma [89]. Emerging modalities, such as bispecific antibodies, are also gaining traction to overcome therapeutic resistance. These agents can simultaneously target multiple antigens or immune checkpoints, enhancing the specificity and effectiveness of immune-mediated tumor cell clearance while minimizing the risk of immune escape [90]. For example, bispecific antibodies targeting LAG-3 and PD-1 have shown promise in enhancing anti-tumor responses through the activation of dendritic cells [91]. The potential for these agents to be combined with established targeted therapies and immunotherapies warrants further investigation to determine their applicability in *BRAF*-mutant melanoma [92]. Moreover, personalized vaccine approaches, particularly neoantigen vaccines tailored to the unique mutation profiles of individual tumors, are emerging as a complementary strategy to bolster anti-tumor immunity. These vaccines have demonstrated encouraging results in early-phase trials across various cancers and could significantly enhance immune responses in patients with *BRAF*-mutant melanoma when used alongside other therapeutic modalities [93]. The integration of artificial intelligence (AI) and machine learning into clinical practice offers additional opportunities for optimizing treatment regimens based on patient-specific data. AI-driven tools can assist in predicting patient responses to therapies, stratifying patients based on molecular and genetic markers, and designing personalized treatment plans that account for the dynamic nature of tumor biology [94]. This technological advancement could lead to improved patient outcomes by facilitating more accurate treatment selections and identifying novel biomarkers for both response and resistance [95,96,97]. Finally, the ongoing exploration of combination therapies, such as *BRAF*/MEK inhibitors paired with novel immune modulators or metabolic reprogramming agents, is crucial for determining their efficacy and safety in patients with *BRAF*-mutant melanoma. Clinical trials that incorporate these emerging therapies in innovative combinations and sequences will be essential in paving the way for more durable responses and extended survival outcomes [86]. Future research should continue to focus on optimizing existing therapies, identifying and validating new therapeutic targets, and developing more precise and personalized approaches to overcome the intrinsic and acquired resistance mechanisms characteristic of *BRAF*-mutant melanoma [87,88].

## 5. Conclusions

Over the past several decades, multiple treatment approaches to managing *BRAF*-mutant melanoma have been developed, including *BRAF* inhibitors, *BRAF*/MEK inhibitor combinations, anti-PD-1 therapy, and anti-CTLA4 therapy. Although they have generally shown marked efficacy in clinical trials, the sustained effectiveness of such therapies has been challenged by changes brought about by and within the TME, which is a highly heterogeneous and complex environment of immunosuppressive cells that collectively affect tumor progression. Although personalized medicine holds great promise for tailoring treatments for *BRAF*-mutant melanomas according to individual genetic profiles, the potential of this approach continues to be challenged by tumor heterogeneity. Through the emergence of this physiological phenomenon, tumors can develop resistance to immunotherapy via various immunological mechanisms, including immune evasion. Moreover, mutations in the *BRAF* gene in melanoma are accompanied by elevated levels of immunosuppressive elements in the immune system, thereby posing an additional challenge to effective personalized immunotherapy for managing the disease. Despite the challenges of the TME, TIL therapy has shown notable efficacy among patients with *BRAF V600*-mutant melanoma, as indicated by the approval of lifileucel as the first TIL therapy for advanced melanoma. By addressing the neoantigen diversity of *BRAF*-mutant melanoma, lifileucel shows substantial promise as an option for tailoring immunotherapeutic treatments for individual patients. Moreover, early response biomarkers such as COX-2 and MMP2 can serve as additional tools for improving personalized immunotherapy by predicting patient responses to melanoma immunotherapy. FDG-PET can be used in conjunction with these biomarkers to identify when immunotherapy should be safely discontinued. To improve the efficiency and patient access to immunotherapy for *BRAF*-mutant melanoma, future efforts should be directed toward shortening T-cell harvesting periods and reducing the costs of TIL therapy.

## Figures and Tables

**Table 1:** Summary of current approaches to personalized immunotherapy for *BRAF*-mutant melanoma.

Type of Approach	Examples	Description of Approach	References
Tumor-infiltrating lymphocyte (TIL) therapy	Adoptive cell therapy, Lifileucel	Isolation of tumor sample via ex vivo expansion and consequent removal of TILs from the tumor microenvironment	[31,32]
T-cell therapy	Chimeric antigen receptor (CAR)-T cell therapy	Genetic modification and tumoral targeting of T-lymphocytes expressing CARs	[58]
Biomarker detection	Circulating tumor DNA, extracellular vesicle-melanoma membrane-bound proteins (LNGFR, MCAM, MCSP, and ERBB3)	Measurement of biomarker levels to predict patient response to immunotherapy or targeted treatment	[47]
Targeted therapy	*BRAF*i, MEKi	Enhancement of personalized immunotherapy using supplementary targeted therapy	[55]
Neoantigens	KRAS G12D	Vaccination with synthesized neoantigen to stimulate a targeted immune response	[63]
